# Deceased donor kidney transplant policies in Asia – implications on practice and recommendations for the future

**DOI:** 10.1016/j.lansea.2023.100312

**Published:** 2023-11-09

**Authors:** Jackson Tan, Muhammad Abdul Mabood Khalil, Terence Kee, Ho Yee Tiong, Taqi Toufeeq Khan, Ihab El-Madhoun, Hideki Ishida, Sanjiv Jasuja, Ghazali Ahmad, Sydney C.W. Tang, Anantharaman Vathsala

**Affiliations:** a PAPRSB Institute of Health Sciences, Universiti Brunei Darussalam, Bandar Seri Begawan, Brunei Darussalam; b King Fahad Armed Forces Hospital, Jeddah, Saudi Arabia; c Singapore General Hospital, Singapore; d National University Hospital, Singapore; e Rehman Medical Institute, Peshawar, Pakistan; f Hamad Medical Corporation, Doha, Qatar; g Tokyo Women’s Medical University Hospital, Tokyo, Japan; h Indraprastha Apollo Hospital, New Delhi, India; i National Heart Institute, Kuala Lumpur, Malaysia; j Department of Medicine, School of Clinical Medicine, University of Hong Kong, Hong Kong; k National University of Singapore, Singapore

**Keywords:** Kidney, Transplant, Asia, Deceased, Cadaveric, Donation, Donor, Policy

## Abstract

Deceased donor kidney transplantation (DDKT) is common in high income Western countries with high transplantation rates. However, the utilization of deceased organs is suboptimal in Asia, due to a multitude of factors. Coherent policies are integral to the development of DDKT programs and deterrence of commercialization, but most are still at an infancy and formative stage in Asia. This review article identifies the glass ceiling effects of social, cultural, religious, political, and technical factors hampering the progress of DDKT in Asia. Additionally, it reviews the history of policy development in different countries and describes their idiosyncratic barriers and challenges. Lastly, it discusses innovative policy measures that can be undertaken to proliferate DDKT practice and curtail commercialization. The long-term ideal is to achieve regional equity and self-sufficiency, through a shared ethos of social and ethical responsibility that transcends and resonates with the different segments of the Asian community.

## Introduction

Asia, as a geographically defined continent, consists of 51 countries in distinct regions broadly classified as West, Central, South, Southeast and East Asia.^1^ End Stage Kidney Disease (ESKD) is on the ascendancy in Asia, with one of the highest incidence rates in the world.^2^^,^^3^ Kidney transplantation (KT) is the preferred treatment of choice for ESKD; with superior long-term survival, quality of life and cost effectiveness compared to dialysis treatment.^4^ The World Health Organization (WHO) has urged countries to progress towards self-sufficiency in transplantation^4^ and emphasized that deceased donor kidney transplantation (DDKT) programs should be high income to its maximum therapeutic potential.^5^

Health policy can transform the way that DDKT program is managed and regulated in a country.^6^ Under an overarching jurisdiction of a policy, engagement with diverse governmental and non-governmental stakeholders can be organized to coordinate and regulate responsibilities pertinent to DDKT. Meticulous and tactful planning is required to facilitate DDKT because it has the potential to stir and evoke a gamut of strong emotional responses from the public; from the depressive lows of sudden bereavements to the regenerative highs of organ donations. Robust preparatory work must be put in to accommodate the requirements of any emergent environment with an inchoate mindset, particularly in Asia, where the community’s sentiments on DDKT are still raw and untested.

Commercialized DDKT is a much discussed and maligned ethical minefield within the transplant community, often exacerbated by the absence of affirmative policies and assertive legislative stance governing community conduct. Recipients often comes from a flawed position of moral high ground, presenting commercialization as a charitable way for the poor to navigate out of poverty. The lack of clarity and transparency in local health policies has led to poor community understanding of the moral principles behind commercialized DDKT, with subsequent proliferation and normalizing of this practice in parts of Asia for many decades.^7^ An important way to curtail commercialization is through achieving organ self-sufficiency through implementation or augmentation of national DDKT programs.

This paper focuses on describing the prevailing DDKT policies and practice in Asian countries and discusses ways to enable the disentanglement of barriers and challenges that impede progress of DDKT in the region.

## Current state of DDKT in Asia

Asia, as a region, has been slow to embrace and utilize KT as the preferred treatment modality for patients with ESKD.^3^ The recent International Society of Nephrology global survey involving 155 countries revealed significant differences in DDKT rates between Asia and high income Western countries, with the disparate rates not merely explained by the income status of countries.^8^ The reported global DDKT median rate in 2018 was 15.1 per million population (pmp); whilst East and North Asia, South East Asia (including Oceania) and West Asia only reported median rates of 3.4 pmp, 6.1 pmp and 4.4 pmp respectively.^8^

South Korea had the highest incidence of KT in Asia with a rate of 44 pmp, with 37% deriving from DDKT in 2021.^2^ East Asia (China, Hong Kong and Taiwan), Iran and Thailand had proportionately more DDKT than Living Donor Kidney Transplantation (LDKT), whilst the rest of Asia had very low rates of DDKT (often < 20% of all KT) in 2021.^2^^,^^9^ Comparatively, high income Western countries had much higher total KT rates (including pre-emptive transplants), with a higher DDKT proportion. United States of America (USA), Spain, UK, Australia and France had KT rates of 75, 73, 54, 44 and 44 pmp respectively in 2021, with 72%, 90%, 73%, 78% and 86% respectively being DDKT.^2^

In 2019, 15 Asian countries contributed data on their DDKT programs to the USRDS^2^ and/or ONT-WHO Global Observatory on Donation and Transplantation.^9^ (Table 1) Additionally, there are case series of DDKT from Sri Lanka,^10^ Vietnam,^11^ Bahrain^12^ and Mongolia^13^ reported in the literature in the last 5 years. International Registry of Organ Donation and Transplant (IRODAT) also reported sporadic DDKT activities in the Philippines, Lebanon and Jordan in the last five years.^14^

**Table 1:** Demographics, treated KRT rates and KT rates of Asian countries with KRT programs reported to USRDS and WHO Transplant Observatory.

Countries	Region	Size (per 1000 sq km)	Population	Treated KRT incidence (pmp)	KT incidence (pmp)	DDKT incidence (pmp)	DDKT/KT %
Saudi Arabia	West Asia	2149	36.4	128	16	2	13
Qatar	West Asia	11.6	2.6	164	18	4	21
Kuwait	West Asia	17.8	4.2	142	32	11	33
UAE	West Asia	83.6	9.4	NR	7	2	29
Iran	Central Asia	1745	88.5	NR	23	15	64
Kazakhstan	Central Asia	2724	19.6	NR	7	1	9
India	South Asia	3287	1425	NR	7	1	11
Singapore	South East Asia	0.7	5.6	337	22	7	33
Thailand	South East Asia	513	71.6	377	11	8	76
Malaysia	South East Asia	330	33.9	262	3	0.5	15
China	East Asia	9562	1425	109	9	8	85
Japan	East Asia	377	125	306	16	2	11
Hong Kong	East Asia	1.1	7.3	179	7	5	73
South Korea	East Asia	100	51.6	360	44	15	35
Taiwan	East Asia	35	23.5	529	18	10	58

Demographics data are obtained from www.worlddata.info.

Treated KRT, KT and DDKT incidence of all countries (except Malaysia, China, Iran, Kazakhstan and UAE) from USRDS 2021.

Treated KRT, KT and DDKT incidence of Malaysia from USRDS 2020

KT and DDKT incidence of China, India, Iran, Kazakhstan and UAE from WHO Transplant Observatory (2019 dataset).

**Fig. 1:**
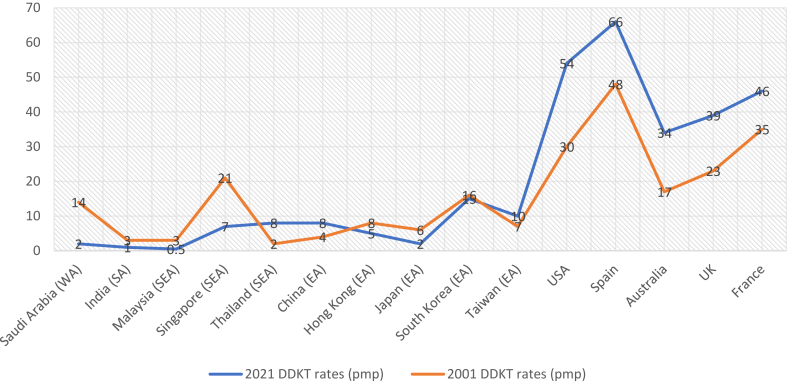
DDKT rates in 2001 and 2021 in selected Asian and Western countries. Abbreviations: WA (West Asia), SA (South Asia), SEA (South East Asia), EA (East Asia), USA (United States of America), UK (United Kingdom). 2021 data was obtained from USRDS and WHO Transplant Observatory. 2001 data from Vathsala et al.

Over the past 20 years, many Asian countries have made poor progress with stagnation of overall KT rates,^15^ despite changes and introduction of different policies and incentives. By contrast, high income countries in the West have made great strides in increasing their KT output, with a greater proportion of organs procured through deceased donation.^15^ Comparing the annual DDKT output over a similar time period (2001 vs 2021), Spain, UK, Australia and France have increased their annual output by 37%, 71%, 102% and 33% respectively.^2^^,^^16^ Some Asian countries, by comparison, have even registered negative growth, possibly from the impact of the criminalization of commercialized DDKT and the ethical position promulgated by the Istanbul declaration.^17^ The 24th Report of the Malaysian Renal Registry reported a drop in overall KT numbers from 131 in 2008 to 82 in 2016, with a corresponding drop of commercialised DDKT numbers from China from 65 to 10.^18^ China, India, the Philippines and Pakistan were the leading destination countries for transplant tourism, with reports of up to 95% of deceased organ donors in China being from prisoners before 2010.^19^ However, commercialized activities in these countries appear to be on the wane after the paradigm shifting initiatives espoused by the Declaration of Istanbul.^17^ Fig. 1 compares the DDKT rates in selected countries in 2001 and 2021.^2^^,^^16^

DDKT graft survival rates in Asia are not dissimilar to those in Western countries, but there is a greater usage of Expanded Criteria Donor (DCD) organs with an older patient demographics in the latter group. Data from the United States Renal Data Registry System showed 1-and 5-year graft survival of 92.7% and 75.6%.^2^ An Iranian study showed 1 year survival of 90.8% for DDKT in 2017, acknowledging a younger donor population from road traffic accidents.^20^ Singapore reported a 1- and 5-year survival of 93.5% and 78.3% from their largest transplant centre in 2020.^21^ Interestingly, data from the Malaysian Renal Registry reported superior commercial deceased donor graft survival from China (1- and 5-year survival of 94% and 84%) compared to their overall deceased donor graft survival (1- and 5-year survival of 84% and 71%).^18^ Hong Kong reported a 10- and 20-year deceased donor graft survival of 70% and 44% in 2018.^22^ The Korean Organ Transplantation Registry from 2022 also showed a similar 1- and 5-year survival of 97.0% and 92.8%.^23^

**Fig. 2:**
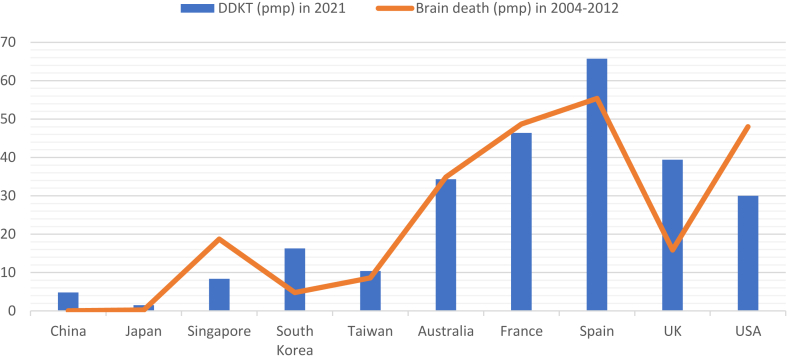
Correlation between DDKT and brain death rates.

The annual brain death rates in United States (48 pmp), United Kingdom (15.9 pmp), Spain (55.4 pmp), France (48.7 pmp) and Australia (34.9pmp) exceeded those in the rest of Asia by a significant margin (Singapore 18.7 pmp, South Korea 4.8 pmp, Japan 0.25 pmp).^24^ China only registered 0.019 brain deaths pmp per year and derived the vast majority of their DDKT from donor after circulatory deaths (DCD).^25^ The reported DCD rates in China and Japan were 2.01 and 0.07 pmp respectively, which paled in comparison with Spain (13.1 pmp), USA (9.82 pmp) and UK (6.5 pmp).^26^ The marked differences could be related to the lack of brain death laws in many countries, with many high income Asian countries only legislating brain deaths in the 1990s (twenty years later than most Western countries). Fig. 2 correlates the DDKT rate in 2021 and brain death rates from data between 2004-2012.^2^^,^^24^

## Why do we need regional policy?

The primary goal of a DDKT policy is to increase rates of KT through regulated and coordinated channels.^6^^,^^27^ Many high income countries have had coherent policies governing KT development for many decades, with recent evolvement of initiatives to prioritize optimal utilization of scarce organ resources whilst maintaining equity in distributions.^28^ This strategy has worked well within a community with a mature and evolved donation mindset, as evident by the increasing numbers of DDKT over the last few decades. The diverse idiosyncratic milieu of Asia calls for adaptation of the different strategies that have been tried and tested in the West, to conform to the level of understanding and acceptance in the region.

Opt-in and opt-out DDKT policies are the two main types of policies that dictate the decision-making process to procure organs after the diagnosis of brain-death.^6^^,^^28^ Opt-in or ‘express consent’ usually requires the donor to have expressed intention to donate through an organ donor register before death. Opt-out or ‘presumed consent’ stipulates that all suitable organs from deceased patients can be procured and allocated to suitable patients on the transplant waiting list. Most countries in Asia will adopt the ‘soft’ opt-in policy, whereby families are still asked for the final donation decision regardless of whether the patients have or have not made their donation intent known beforehand.^6^ Contextually this is important in Asia, where the accepted maxim is for family to be involved with decisions regarding preservation of posthumous dignity, regardless of prevailing legal policy jurisdiction. Mandated organ donation policy, usually at the time of application for official government documentation like passport or driving license applications, on the donation preference of individuals is practiced by many countries with an opt-in policy.^6^^,^^28^

The call for the creation and strengthening of policies to maximize the utility of deceased donor organs is particularly more desperate in Asia, given the soaring incident and prevalent rates of ESKD in the region.^3^ More efforts are needed to build and assemble an ancillary network of support system to complement existing policies. The need is particularly urgent in lower income countries, due to the absence of national policies to govern and give direction to local KT activities, nurturing a potential environment for illegal proliferation of commercialized activities and exploitation of vulnerable communities.

## Legislative milestones, history and policy directions in Asian countries

Although KT activities had started in the 1960s in Asia,^16^ many countries do not have official legislative endorsements until many years or decades after. Ad-hoc transplant activities were performed within the confines of local guidelines, with guidance and inputs from pioneering experts and foreign policies. As experience accumulated in subsequent years and through gradual transformative education of the involved community, legislated governance of program becomes necessary; particularly to publicize national stances on commercialization, gazette penal offences, strengthen consenting procedures and improve procurement and allocation regulations.

Taiwan was the first country in Asia to have a human organ transplant act in 1985, 17 years after the maiden kidney transplant operation was performed in the country.^29^ Singapore reported their first DDKT in 1970 and adopted an ‘opt-in’ transplant policy in 1973, which was later supplemented to ‘opt-out’ in 1987 following the formalization of their Human Organ Transplant Act.^21^ As a way of encouraging donation, the Singapore Government also supports deceased donor families by reimbursing costs of the terminal hospitalization directly to the donor hospital and maintaining cost neutrality for donor families.^21^ Malaysia, Vietnam, Hong Kong, Japan and South Korea attained government-approved organ donation legislation in 1974, 1992, 1996, 1997 and 2000 respectively.^11^^,^^30^, ^31^, ^32^, ^33^

China passed their first law of organ transplantation in 2007 with six integral features: nationwide system, legal framework criminalizing commercialization, regulatory mechanisms, categorizing quality of cadaveric organs, focus on humanitarian assistance through socialist policy and embracement of humanistic spirit that embodies traditional virtues.^34^ This pivotal move has had some effect in reducing unethical organ procurement from prisoners for transplantations from many East and South East Asian countries. In a similar move with significant repercussions for commercialization, but not so much for DDKT, India enacted its national organ transplant act in 2011 with specific deceased donor considerations to facilitate and coordinate activities in the country.^35^ The National Organ and Tissue Transplant Organisation in India provides financial support for management of potential deceased donors in retrieval centres, provides free registration for retrieval centres and financial assistance to transplant recipients below the poverty line.^36^

In Iran, religious authorization through fatwa was passed in the 1990s, but deceased donor numbers only increased after legislation to permit DDKT was passed in 2000.^37^ Kuwait, Oman, Qatar, United Arab Emirates and Saudi Arabia enacted their transplant laws in 1987, 1996, 1997, 2017 and 2021 respectively.^38^, ^39^, ^40^, ^41^, ^42^ To bolster donation, Saudi Arabia compensates the deceased donor’s family 50,000 riyals and gives 50% discount for travels on Saudi Airlines.^42^ Qatar, Kuwait and UAE also provide financial incentives to family and have unique schemes which allow expatriates to contribute towards and receive DDKT.^40^

Brain death law was also absent in many Asian countries, until the 1990s when definitive legislations were issued in Malaysia, Indonesia, Thailand, Japan, Singapore and South Korea.^24^ There is currently no brain death legislation in China with no national medical standards for making the diagnosis.^24^ Brain death law is evident in Middle Eastern countries, although exact criteria for diagnosis may vary between countries.^43^ However, there is still a sizeable minority in the Muslim world that only accepts death by cardiopulmonary criteria.^43^ Table 2 summarises the demographics, legislative milestones and policy directions of Asian countries.

**Table 2:** Demographics, legislative milestones and policy directions of Asian countries.

Country	Region	Legislated policy	Legislative policy measures to improve deceased donation
China^34^^,^^44^	East Asia	The first law of organ transplantation was passed in 2007 with six integral features: nationwide system, legal framework criminalizing commercialization, regulatory mechanisms, categorizing quality of deceased donor organs, focus on humanitarian assistance through socialist policy and embracement of humanistic spirit that embodies traditional virtues	Transplant tourism was officially prohibited in 2007 and the act of commercialization is criminalised by law in 2011. The Chinese Government initiated the Donation after Citizens’ death policy in 2010, which was promoted nationwide after a 3-year implementation pilot. To regulate the rapid increase in the quantity of KT, China has set up organ transplant quality control centres in 2016 to supervise, monitor and inspect transplant programs. Furthermore, the Clinical Guideline for Organ Transplantation in China was published in 2018 to improve the quality of clinical care.
Japan^31^	East Asia	The organ transplant law took effect in 1997, but due to the stringent opt-in policy, the numbers were limited. This was revised in 2010	The revision in 2010 allows for the removal of organs from patients with the consent of only bereaved family and the use of extended criteria donors.
South Korea^32^^,^^45^	East Asia	The organ transplant act was enacted in 2000, with amendments in 2002, 2006 and 2010 to incorporate changes designed to increase donations.	The organ incentive system was introduced to give priority to relatives of the deceased and the proximity (hospital or region) of the potential recipient to the donor. Further efforts to increase donations are made through initiatives like involvement of intensive care and emergency department personnels for early identification of brain-death, reducing family refusal through ‘my family should know my wish’ campaign, high school and middle school education program and objective monitoring of transplant status through registry network.
Hong Kong^30^	East Asia	The Human Organ Transplant Ordinance was inaugurated in 1996, with the latest amendments in 2020.	The Department of Health administers a Centralised Organ Donation Register for prospective donors to indicate their wish to donate organs after death. An organ transplant mutual assistance mechanism between HK and mainland China is under planning.
Taiwan^46^	East Asia	Taiwan was the first country in Asia to issue a human organ transplantation act in 1987 to regulate organ transplant.	Taiwan organ registry and sharing centre was later established in 2005 to serve as a bridge between patients, procurement hospitals and organ transplantation hospitals.
Mongolia^13^	East Asia	Mongolia’s donor law was approved in 2000, with updates in 2012 and 2018 to revise the donor bill to legalise organ donation after brain death	The Government approved fundings for organ transplant to reduce out of pocket costs for patients in 2017. Transplant hospitals also compensated deceased organ-related costs through the general transplant budget. Additionally, the transplant team was sent for specialized funded training in South Korea, United Kingdom, India and the United States.
Singapore^21^	Southeast Asia	The Human Organ Transplant Act was inaugurated in 1987. However, ‘opt-in’ policy was operational from 1973, which was subsequently changed to ‘opt-out’ in 1987 with amendments made in 2008 to increase the donor pool.	Inclusion of Muslim patients as opt-out and marginal organs in 2009. Re-imbursement to donor hospital for terminal hospitalisation bill in relation to pre-donation care and 50% subsidy of some medical expenses at public hospitals for 5 years for immediate family members of deceased donors.
Malaysia^47^	Southeast Asia	The Human Tissues Act was established in 1974 but only relates to deceased donors, whereas live donors fall within the purview of the common law. The National Organ Tissue and Cell Transplantation Policy was issued in 2007.	A National Transplant Resource Centre under Ministry of Health maintains a database on organ pledgers, organizes and coordinates national organ donation promotion activities and manages national procurement activities for organs and tissues. It also helps with deceased donor allocation and national transplant list managed by the Ministry of Health
Thailand^48^	Southeast Asia	No specific law but only the basic principles provided by Medical Council and the Red Cross (NGO).	The Organ Donation Centre is a non-governmental organization (Red Cross) is responsible for national waiting lists, centralized deceased donor allocation. National insurance policy through various schemes recently extended universal health coverage that extends to peritoneal dialysis and transplant patients.
Vietnam^49^	Southeast Asia	The law on donation, removal and transplantation of human organ and donation was enacted in 1992. Amendment and supplementation of the law were made in 2001.	Transplant team was sent for training in Europe, Japan, South Korea and Taiwan to obtain clinical experience and expertise.
India^35^^,^^36^^,^^50^	South Asia	Transplantation of human organs (amendment) Act was enacted in 1994 to provide a system of removal, storage and transplantation of human organs for therapeutic purposes. It was amended in 2011 with specific deceased donor considerations.	The 2011 amendments allow for registration and provision of retrieval centres, provision of mandatory transplant coordinators in hospitals, simplification of brain death certification, mandatory enquiry from attendants of deceased to potential donor family about permission to donate. The National Organ and Tissue Transplant Organisation (NOTTO) provides financial support for management of potential deceased donors in retrieval centres and financial assistance to transplant recipients below the poverty line. Centres can also be registered for organ retrieval without any cost in areas without transplant centres. NOTTO also works at state (SOTTO) and regional level (ROTTO) for organ allocation and maintenance of waitlist.
Sri Lanka^51^	South Asia	Organ donation in Sri Lanka was ratified by the Human Tissue Transplantation Act in 1987.	A national strategic plan (2022–2026) to promote DDKT was launched through developing national and subnational level institutional mechanism, strengthening human resources, transfusion services and laboratory services. Promotion of transplant-related research, collaboration with international organisations and community empowerment were also key strategies that were mentioned.
Iran^37^^,^^52^	Central Asia	Religious authorization through fatwa was passed in the 1990s, but deceased donor numbers only increased after the Organ Transplantation Brain Death Act was passed to permit DDKT in 2000	The Iranian model in KT created short waiting list for LKT through both related and unrelated means, which has positive impact on decreasing commercialization through black market. The government has allocated national funds to organ procurement units according to number of organs procured. Shiraz,the largest DDKT program in Iran, deters living unrelated kidney transplant through imposing 6-month compulsory waiting list. Partnership with local leaders and universities to promote education and awareness have also built trust within the community.
Saudi Arabia^42^^,^^43^	West Asia	A law on The Human Organ Donation passed in 2021 aims at streamlining the process of organ donation and obtaining permits to donate organs after death.	The National Kidney Foundation was established in 1985 to implement and coordinate a local deceased organ transplant program. This evolves to become the Saud Centre for Organ Transplantation (SCOT) to supervise and regulate transplant activities and promote awareness in the country. SCOT also heads and participates in organ sharing network in the Arabian Gulf Region through the Gulf Health Council. The government compensates the deceased donor’s family 50,000 riyals and 50% discount of Saudi Airlines.
Qatar^43^	West Asia	Organ transplantation was legalized in 1997 through an Amiri decree on regulation of human organ transfer and transplantation.	The Doha model introduced in 2011 focus on equity of organ access to all residents, regardless of citizen status, through a multilingual and multicultural education approach to engage the diverse communities in Qatar. It also participates in the Gulf Health Council sharing scheme. Travel and counseling support is provided for the deceased donor’s family.
UAE^41^^,^^53^	West Asia	The legal framework for deceased donor kidney transplantation was created in 2017, which confirms the legal definition of brain death, hence allowing DDKT.	Cleveland Clinic Abu Dhabi provides clinical expertise in complex and multi-organ transplants and shares clinical insight and best practice with SCOT. It also participates in the Gulf Health Council sharing scheme A national centre for regulating human organs and tissues transplantation was established in 2020 to unify and coordinate national efforts across the country. In addition, any person over the age of 21, regardless of nationality, can register intention to donate after brain death.
Kuwait^38^^,^^53^	West Asia	Organ transplant legislation was established in 1987, with deceased donor program officially started in 1996.	It participates in the Gulf Health Council sharing scheme. Incentives are given to donor’s families, whether locals or expatriates, after DDKT. Support for expatriates includes repatriation of body back to their home countries at the expense of the Ministry of Health. In addition, financial support are given to the deceased donor’s family for other medical expenses if they are poor.
Oman^39^	West Asia	A Royal Decree in 1996 allows organ donation after brain death under certain circumstances and in accordance with religious and medical practices. A Fatwa issued by the Grand Mufti of Oman endorsed the decision to permit organ donation under the jurisdiction of Shariah law in 2014.	In 2018, the Ministry of Health issued guidances for human organ and tissue transplantation with emphasis on developing a regulatory national program to oversee activities in private and public hospitals. Approval was also given for transplant donation to distant relatives (up to fourth degree).

All data from this table are obtained from the quoted references from published literature and governmental websites. Additional countries’ information are provided by the co-authors of the manuscript.

## Barriers and challenges with different transplant policies

Achieving an equilibrium for optimal utility of scarce organ resources within the complex socio-cultural environment in Asia is challenging. Barriers and challenges reported in high income Western countries may not be applicable, because Asian countries have unique population demographics with diverse ethnic and cultural heterogeneity. There is also an extreme disparity in national population, geographical sizes, income status and health funding mechanisms in the region.^1^^,^^54^

### Cultural influences

Asia has proportionately less female DDKT recipients; indicating gender inequality in access to KRT.^55^ The diverse racial heterogeneity of the Asian population also means that any policy direction must incorporate the viewpoints and buy-in potential of the different ethnic and cultural subgroups of the population. Cultural adaptations must be made to allow changes to procurement protocol.^56^ Cultural and religious practices often intertwine in East Asia; with the majority of the population following the teachings of a quasi-religious hybrid of Confucianism, Buddhism, Taoism and Shintoism, alongside cultural-influenced ancestral worship. Confucianism principles suggest that KT should be prioritized to close relatives or people of same ethnic groups, as opposed to strangers.^56^ Some Buddhists believe that the dying process may take several hours (or even days) and organs can only be removed after a specified period has passed, hence affecting the viability and useability of the organs.^24^ Followers of the Taoist faith may believe that only certain organs can be removed for donation, due to major organs having a relationship with nature.^24^ Shintoism traditions, bound by the idea of purity and wholeness of the physical body, state that interfering with a corpse brings bad luck and may injure the relationship between the dead person and the bereaved.^57^

### Religious influences

In Islam, the interpretation and application of the concept of darurah (necessity) and deliberation in the formulation of fatwas (formal ruling by Islamic law) on DDKT differs amongst different Muslim countries and Islamic scholars.^43^ Delaying funeral for deceased donation may cause many families to refuse kidney donation amongst Muslim communities.^56^ Definition of neurological or brain death is still unclear in certain segments of the Islamic community, due to concerns that the term is not well defined and the inability of modern technology to definitively diagnose irreversible cessation of brain function.^58^ Some Hindus may also believe that deceased donations might interfere with the traditional incineration of corpses and fear that bad karma may be transferred from the deceased to the recipient.^57^

### Poor public knowledge and attitude

Poor health literacy and inadequacy of the education system in Asia mean that most of the population are unaware of the benefits of transplantation. The concept of brain death and organ donation is poorly understood, which results in many families refusing donation when approached.^8^ Studies have shown that Asian patients residing in UK and USA had poorer knowledge and pervasive attitude towards DDKT.^59^^,^^60^ Local studies from the Saudi Arabia, India and China have echoed these findings, through a wide range of respondents; from the general public to healthcare personnels.^61^, ^62^, ^63^

Mistrust in public services in Asia may also be a factor that undermines deceased donation, primarily towards determination of death and process of organ procurement. There is a misconception that the health profession may get financial benefits and will not do diligent service to save the life of a patient in order to facilitate organ transplant. Rural communities with substantial indigenous population may also have a poor acceptance of modern medicine and healthcare system.

### Technical and financial support

A viable DDKT program must be backed by substantial financial and technical support, alongside experienced expertise. This includes having the proficiency to set up legal framework in the country, promoting public education, training of medical and nursing personnel, setting up laboratory services for cross-matching, educating personnel in intensive care and high dependency units to identify and report potential candidates and setting up a donor registry system within the country. Engagement of foreign experts through international bodies is often necessary to kickstart the process. Opportunities to partner established institutions in high income countries may also be limited by financial constraints. The disparity in healthcare access in Asia means that higher income countries and those with universal health care or access to insurance have higher transplantation rates compared to less affluent countries.^54^ ESKD patients in the latter group will need to make ‘out of pocket’ payments for transplant care and medications, making them less likely to seek treatment.

### Availability/acceptability of commercialization

The recent Declaration of Istanbul has enabled many countries to understand the principles of ethical transplant and take a stance against commercialization. However, there has been mass and social media reports of an increase in illegal transplant activities in developing countries following the long travel hiatus caused by the COVID-19 pandemic, although this has yet been verified in peer-reviewed medical literature. The persistent presence of these commercialized activities will continue to hamper public trust and negate the urgent need to establish legitimate, self-sufficient local program.

### Wastage of organs

There is a significant disparity between the rates of brain death diagnosis between Asia and high income Western countries.^24^ This indicate that many brain death patients are not picked up in intensive care units or there is a failure of communication with key hospital personnel to indicate the availability of potential candidates. There is limited prevalence data in the literature on the usage of ECD organs in Asia, perhaps indicating the deficiency of such practice, but the Korea Network for Organ Sharing reported a commendable 35% of DDKT being derived from ECD between 2005 to 2014.^64^ China also reported that 67% of all deceased donors were from circulatory deaths in 2019.^9^

## Recommendations for future policy developments

Variations in transplant policy and practice in many Asian countries have affected access to transplantation, especially in lower income countries. A multi-stakeholder approach should be undertaken at different levels within the society—international, national, sub-national and institutional. A similar approach has been implemented in the European Union (Roadmap) to bolster transplant productivity in an already mature and developed system by enhancing equity and improving rates in weaker member nations.^28^ An European Union action plan (2009–2015) had already increased number of KT in member nations by 15% between 2010 to 2019.^65^ The number of European countries with DCD programs also increased from 10 to 28.^66^ A similar stimulatory intervention exercise can also be conducted in Asia to galvanise DDKT programs in the region. The different recommendations stratified according to population levels are summarized in Table 3.

**Table 3:** Recommendations to improve DDKT at stratified population levels.

Levels	Description	Potential actors/stakeholders
International	Policy sharing between countries	World Health Organisation
	Visiting international expertise through forums, workshops or partner hospital arrangements	Asian Society of Transplantation International Society of Nephrology
	Organ donor registry with potential to share organs between neighbouring countries	Gulf Health Council Association of Society of South East Asian Nations (ASEAN)
	Financial support for low-income countries	International non-governmental organisations and charities
	International position statement from religious bodies and transplant organisation	Organisation of Islamic Cooperation The Transplantation Society
National	Legislation on brain death	Local government (law)
	Legislation on commercialisation	Local government (law)
	Improving public education and awareness through school curriculum	Local government (education, home affairs)
	Incentives and reimbursements to family	Local government (finance, health) Non-governmental organisations (charities)
	Financial support for recipients for hospital costs and maintenance medication	Local government (finance, health) Non-governmental (charities, insurance and pharmaceutical companies)
	National-level coordination bodies for procurement, allocation and hospital services	Local government (home affairs, health)
	Consider the merits of opt-out policy	Local government (law, health)
	Mandated donation policy	Local government (law, home affairs)
Sub-national (state, province and district)	Engaging religious leaders/public figures/politicians	Suitable candidates with knowledge and clout in community
	Streamlining procurement, allocation and hospital services	Community-level coordination bodies
	Improving community perception of transplant, donation and brain death	Mass and social media
	Research to assess barriers, improve efficacy and investigate benefits of program	Universities Pharmaceutical companies
Institutional	Improve awareness and skills amongst healthcare personnels	Hospital and allied healthcare services
	Improve hospital capabilities	Hospital services
	Multispecialty coordination within the transplant hospital	Hospital services
	Improve technical expertise of physicians, surgeons, coordinators and nurses	Individual
	Retention of key personnels and pioneers to avoid brain drain	Individual

### International

Policy sharing and reporting mechanism through regional organ donor registries should be fostered, ideally through the formation of clusters according to geopolitical alliances.^67^ Eurotransplant is a successful model that is responsible for the allocation of organs to 8 European countries since the 1960s.^68^ This process will help smaller and low income countries develop faster by taking on apprenticeship roles. Currently, Hong Kong and China have discussed an organ donation scheme that can provide patients with high immunological risk a better chance of getting a suitable match.^69^ A similar initiative is in place in the Middle East, with the Saudi Centre of Organ Transplant (SCOT) heading an organ and expertise sharing network in the Arabian Gulf Region through the Gulf Health Council.^42^ Organized networks between countries could initially focus on capacity building to strengthen existing frameworks in small and developing countries, before instituting deceased organ exchange schemes which may be challenging given the geographical distances between countries in Asia.

Major health, nephrology and transplant organizations can also work with local pioneers in smaller countries to develop and accelerate the necessary processes through forums, workshop and establishing partnership relationships. International non-governmental organizations and transplant-related pharmaceutical companies can provide monetary support to kickstart DDKT activities. More assertive stances could be publicized by international religious organizations to endorse donation and provide clarity about their position with brain death.

### National

There should be government-led governance of DDKT program in low- to middle-income countries due to the fragmented and poorly-funded healthcare systems, establishing clarity with the roles of secondary (procurement and allocation) and tertiary care (transplant hospital) to streamline DDKT activities. Government-led policies are needed to coordinate multi-sectorial contributions from different governmental agencies organizations, particularly to strengthen the legal framework for brain death diagnosis and penalize commercialization. Educational activities on organ donation must be inculcated at grassroot levels, to groom the mindsets of the future generations.

Mandated transplant donation policy should be introduced for the public to register donation intent, but most importantly to get the community to initiate and engage in a dialogue about transplant. Financial reimbursement systems may work better in the Asian environment, where the concept of altruism alone may not be enough to sway family’s reluctance to consent for donation. Many patients in lower- and middle- countries in Asia, with no access to welfare and insurance system, will need financial support to afford transplant care.

### Sub-national (state, provinces, and districts)

Suitable candidates with knowledge and clout in community like religious leaders and public figures should be engaged to broadcast useful information to their local communities. Research, through universities, on nuanced local needs especially on health economic comparison of DDKT programs and barriers in different countries are necessary to assess feasibility and sustainability. To align with contemporary technological and gadgetry trends, it is important to establish a working relationship with social media influencers and journalists to reach out to tech-savvy and younger generations in the community.

In larger countries, community- or state-level coordination bodies (either government or non-government entities) may be more effective in coordinating the efficient utilization of local deceased donor organs, with priorities given to the immediate and preferred needs of the local community. Two successful examples of sub-national models are the Tamil Nadu state in India and Shiraz city in Iran. The Tamil Nadu state, a leader in deceased organ donation in India, provides free transplantation facilities to the underprivileged population through public-private enterprises with procurement of generic medications and provision of specialized bereavement training for transplant coordinators through the Multi-Organ Harvesting and Network (MOHAN) foundation.^70^ The city of Shiraz has the largest DDKT program in Iran, with 87% of KT being from deceased donors. The program deters living unrelated kidney transplant by imposing a compulsory 6-month wait and working with local leaders to promote deceased donation in mosques and public venues.^52^

### Institutional

Improving education and awareness of key hospital staff is crucial, especially for frontliners (intensive care doctors and transplant coordinators) that initiate the process of donation. Training is needed to ensure that wastage from declined consent is kept to a minimum, through consideration of organ donation routinely in all end-of-life discussions with patients and relatives. Coordinated institutional efforts can minimize the duration of the donation process to avoid lapse of the ‘golden period’ through prompt identification of potential candidates, early diagnosis of death, skillful negotiations with family and maintenance of haemodynamic status of donor. The hospital must be properly equipped with enough operating theatres, specific laboratory capabilities, adequate nursing staff and skilled doctors; for rapid activation of transplant processes upon the identification of potential candidates. In countries where there may be nebulous religious agreement on brain death, transplantation through DCD should be prioritized.

Advances in organ perfusion protocols can prolong viability of organs and adopting risk scoring and histology-based classifications of expanded criteria organs can minimize wastages.^71^ Refining surgical techniques through transplanting both kidneys from an ECD to a recipient can result in efficient utilization of organs. Countries with low volume of transplant surgeries must retain qualified medical personnels to avoid ‘brain drain’ to high income countries with larger transplant centres, which can jeopardise the development and sustainability of established program.

### Limitations

This review is guided by published literature, collective expertise of authors, national registries and governmental websites. Literature review was conducted in the English language through Google Scholar, Pubmed and Scopus; data that are presented in other languages may be missed, resulting in a small information gap. Asian countries with DDKT programs and geopolitical affiliations with Europe (Russia, Turkey, Israel and Azerbaijan) are excluded from this review.

We also acknowledge that an exact temporal comparison between incidence rates is often not possible due to the asynchronous data from different sources. Many Asian countries also do not have publicly accessible registries or registries that publish data consistently, making time-aligned comparisons difficult. The COVID-19 pandemic has affected the availability and reliability of data between 2020 and 2022. KT program activities were also disrupted during this period. Therefore, data used from this review were deliberately obtained from the pre-pandemic era (before 2020), which should give a better representation of KT activities.

## Conclusion

This review indicates that DDKT policies in Asia are still at an early developmental stage, but play a crucial role in guiding and driving activities in Asian countries. Higher income countries in Asia have a greater capacity to develop successful policies to establish framework for DDKT, albeit still not as effectual as some of the countries in the West. The success of Asian DDKT programs will depend on the efficacy of local polices to strengthen local transplant infrastructure, improve public engagement, coordinate multi-sectorial support and establish legislative jurisdictions. Comprehensible and realistic approaches are required to coordinate efforts and bridge the chasm with lower income countries, particularly to curb commercialization. The long-term ambition should be to enable regional equity and self-sufficiency through a shared ethos of social and ethical responsibility, that transcends and resonates with the different segments of the Asian community.

## Contributors

Jackson Tan researched the literature and wrote the first draft of the manuscript. All authors reviewed and edited the manuscript and approved the final version of the manuscript.

## Declaration of interests

None of the authors declare any conflict of interest. This project did not receive any fundings.

## References

[1] Countries’ data from information from. https://www.worlddata.info/asia.

[2] Information from the United States renal data system at. https://usrds-adr.niddk.nih.gov.

[3] Liyanage T., Toyama T., Hockham C. (2022). Prevalence of chronic kidney disease in Asia: a systematic review and analysis. BMJ Glob Health.

[4] Delmonico F.L., Dominhuez-Gil B., Matesanz R., Noel L. (2011). A call for government accountability to achieve national sufficiency in organ donation and transplantation. Lancet.

[5] WHO, Transplantation Society (2011). Third WHO global consultation on organ donation and transplantation: striving to achieve self-sufficiency, March 23-25, 2010, Madrid, Spain. Transplantation.

[6] Etheredge H.R. (2021). Assessing global organ donation policies: opt-in vs Opt-out. Risk Manag Heathc Policy.

[7] Rahbi F.A., Salmi I.A. (2017). Commercial kidney transplantation: attitude, knowledge, perception, and experience of recipients. Kidney Int Rep.

[8] Mudiayi D., Shojai S., Okpechi I., Christie E.A., Wen K., Kamaleldin M. (2022). Global estimates of capacity for kidney transplantation in world countries and regions. Transplantation.

[9] Information from. https://www.trasnplant-observatory.org/.

[10] Ahamed M.M.S.B., Latif M.N.A. (2023). Deceased donor kidney transplantation outcomes at a Sri Lankan center: a comprehensive single-center analysis. Cureus.

[11] Nguyen H., Bich H.T.T., Ngoc T.D.T. (2022). Outcomes of deceased donor kidney transplantation at cho ray hospital, Vietnam. Kidney Int Rep.

[12] Khder M.A., Noaimi A.L. (2019). Organ donation in Bahrain. Transplant Res Risk Manag.

[13] Gonchigjav B., Bayaraa A., Batjargal E. (2022). Organ donation and transplantation in Mongolia. Transplantation.

[14] Information from the international registry on organ donation and transplantation at. https://www.irodat.org.

[15] Wu D.A., Watson C.J., Bradley J.A., Johnson R.J., Forsythe J.L., Oniscu G.C. (2017). Global trends and challenges in deceased donor kidney allocation. Kidney Int.

[16] Vathsala A. (2004). Improving cadaveric organ donation rates in kidney and liver transplantation in Asia. Transplant Proc.

[17] The declaration of Istanbul. https://www.declarationofistanbul.org.

[18] Information from the Malaysian renal registry. https://www.msn.org.my/nrr.

[19] Biggins S., Bambha K., Terrault N., Inadomi J., Roberts J.P., Bass N. (2009). Transplant tourism to China: the impact on domestic patient care decisions. Clin Transplant.

[20] Rezapour S., Yarmphammadi A., Tavakkoli M. (2017). One-year survival rate of renal transplant: factors influencing the outcome. Transpl Res Risk Manag.

[21] Kee T. (2020). Renal transplantation at the Singapore General Hospital: a miracle in the making over 50 years. Proc Singapore Healthc.

[22] Chau K.F. (2018). Living-related renal transplantation in Hong Kong. Hong Kong Med J.

[23] Jeon H.J., Koo T.Y., Ju M.K. (2022). The Korean Organ Transplantation Registry (KOTRY): an overview and summary of the kidney transplant cohort. Kidney Res Clin Pract.

[24] Yang Q., Miller G. (2015). East-West differences in perception of brain death. J Bioeth Inq.

[25] Zhou J.S., Dou K.F. (2011). Confusion and hopes for organ transplant in China.Organ. Transplantation.

[26] Kaposztas Z. (2022). Donation after circulatory death- activity review in the Asian region. Transplant Rep.

[27] Godwin M. (2022). Nephrology policy: kidney transplantation. Adv Chronic Dis.

[28] Vanholder R., Dominguez-Gil B., Busic M. (2021). Organ donation and transplantation: a multi-stakeholder call to action. Nature.

[29] Lee C.J. (1996). Organ transplantation in Taiwan. Artif Organs.

[30] Fan R.P., Chan H.M. (2017). Opt-in or opt-out: that is not the question. Hong Kong Med J.

[31] The Japan Organ Transplant network http://www.jotnw.or.jp/en/04.

[32] Joo H.N. (2013). The organ transplantation act and recent trends in Korea. Asia Pac J Publ Health.

[33] (2005). Organ transplantation in Malaysia: a need for a comprehensive legal regime. Med Law.

[34] Huang J. (2017). The “Chinese mode” of organ donation and transplantation. Hepatobiliary Surg Nutr.

[35] Directorate General of Health Services, India https://dghs.gov.in/content/135.

[36] Kute V., Vasanthi R., Rela M. (2021). On the way to self-sufficiency: improving deceased organ donation in India. Transplantation.

[37] Abbaszadeh S. Nourbala MH, Taheri S, Ashraf A, Einollahi B. Renal transplantation from deceased donors in Iran. Saudi J Kidney Dis Transpl. 2008;19(4):664–668. 18580034

[38] Jamal M. (2014). The status of organ transplantation in Kuwait. J Kuwait Med Assoc.

[39] Information from. https://royalhospital.med.om/eng/organ-transplant.php.

[40] Martin D., Fadhil R. (2014). The Doha model of organ donation and transplantation: thinking beyond citizenship. Griffith J Law Hum Dignity.

[41] Kumar S. Sankari BR, Miller CM, Al Obaidli AAK, Suri RM. Establishment of solid organ transplantation in the United Arab Emirates. Transplantation. 2020;104:659–663. 10.1097/TP.0000000000003030 32224809

[42] Shaheen F., Souqiyyeh M.Z., Al-Swailem A.R. (1995). Saudi center for organ transplantation: activities and achievements. Saudi J Kidney Dis Transpl.

[43] AlSulaiman N.S., Alassaf M.A., Boumarah D.N., Almubireek A.M., Alkaltham G.K., Menezes R.G. (2021). Organ transplantation in Arabian Gulf countries: ethical and legal practice and beyond. Forensic Sci Med Pathol.

[44] Shi B.Y., Liu Z.J., Yu T. (2020). Development of the organ donation and transplantation system in China. Chinese Med J.

[45] Ahn C., Lee S., Kim Y.H. (2021). Improving self-sufficiency in organ transplantation in Korea. Korean J Transplant.

[46] Wang T.H., Chang Y.P., Chinag W.L. (2016). Improving donation rates in Taiwan. Transplantation.

[47] Ministry of Health Malaysia, Medical development Division (2016). The national organ Tissue and cell transplantation policy. Putrajaya. http://www.moh.gov.my/images/gallery/Polisi/National_Organ_Tissue_%20Cell_Transplan_Policy.pdf.

[48] Nivatvongs S., Dhitavat V., Jungsangasom A. (2008). Thirteen years of the Thai red cross organ donation centre. Transplant Proc.

[49] Information from the official Vietnam government website @. https://vbpl.vn/boyte/Pages/vbpqen-toanvan.aspx?ItemID=3792.

[50] Kute V.B., Meshram H.S., Mahillo B., Domínguez-Gil B. (2023). Current status, challenges, and opportunities of organ donation and transplantation in India. Transplantation.

[51] Information from. https://nationaltransplant.health.gov.lk/.

[52] Ghahramani N. (2016). Paid living donation and growth of deceased donor programs. Transplantation.

[53] Ali A., Hendaway A. (2015). Renal transplantation in the Middle East: strengths, weaknesses, opportunities and threats [SWOT] analysis. Urol Nephrol Open Access J.

[54] Tang S.C.W., Yu X., Chen H.C. (2000). Dialysis care and dialysis funding in Asia. Am J Kidney Dis.

[55] Han M., Wong G., Kute V.B. (2023). Gender disparity in Asian-Pacific countries: an analysis of the ASTREG-WIT-KT registry. Transplantation.

[56] Robson N. (2010). Organ transplants: ethical, social and religious issues in a multicultural society. Asia Pac J Public Health.

[57] Doerry K., Oh J., Vincent D., Fischer L., Schulz-Jurgensen S. (2022). Religious and cultural aspects of organ donation: narrowing the gap through understanding different religious beliefs. Pediatr Transplant.

[58] Miller A. Ziad-Miller A, Elamin EM. Brain death and Islam: the interface of religion, culture, history, law and modern medicine. Chest. 2013;146(4):1092–1101. 10.1378/chest.14-0130 PMC4188144 25287999

[59] Cheung A.H.S., Alden D.L. (1998). Wheeler MS.Cultural attitudes of Asian-Americans toward death adversely impact organ donation. Transplant Proc.

[60] Randhawa G. (1998). The impending kidney transplant crisis for the Asian population in the UK. Publ Health.

[61] Sharaan R., Alsulami R., Arab R. (2021). Knowledge, attitude and willingness toward kidney donation among health sciences students at King Saud bin Abdulaziz University. Front Public Health.

[62] Sarveswaran G., Sakthivel M.N., Krishnamoorthy Y., Arivarasan Y., Ramakrishnan J. (2018). Knowledge, attitude and practice regarding organ donation among adult population of urban Puducherry, South India. J Educ Health Promot.

[63] Fan X., Li M., Rolker H. (2022). Knowledge, attitudes and willingness to organ donation among the general public: a cross-sectional study in China. BMC Publ Health.

[64] Song S.H., Lim S.H., Lee J. (2018). Impact of Korea network for organ sharing expanded donor criteria on delayed graft function in kidney transplantation: a single-center experience. Transplant Proc.

[65] Vanholder R., Stel V.S., Jager K.J. (2019). How to increase kidney transplant activity throughout Europe-an advocacy review by the European Kidney. Health Alliance.

[66] Lomero M., Gardiner D., Coll E. (2020). Donation after circulatory death today: an updated overview of the European landscape. Transpl Int.

[67] Tan J., Liew A., Koh D. (2022). Clinical outcomes and performance indicators of patients with kidney failure and acute kidney injuries in ASEAN countries. Nephrology (Carlton).

[68] Putzer G., Gasteiger L., Mathis S. (2022). Solid organ donation and transplantation activity in the Eurotransplant area during the first year of COVID-19. Transplantation.

[69] Information from. https://www.youtube.com/watch?v=2t35oktCEhA.

[70] Abraham G., Vijayan M., Gopalakrishnan N. (2016). State of deceased donor transplantation in India: a model for developing countries around the world. World J Transplant.

[71] Smith M., Dominguez-Gil B., Greer D.M., Manara A.R., Souter M.J. (2019). Organ donation after circulatory death: current status and future potential. Intensive Care Med.

